# Effects of levothyroxine replacement therapy on insulin resistance in patients with untreated primary hypothyroidism

**DOI:** 10.1186/s13104-023-06516-7

**Published:** 2023-09-29

**Authors:** Alireza Ostadrahimi, Taher Manzari, Sahar Gohari-Lasaki, Helda Tutunchi, Majid Mobasseri, Vahideh Sadra, Farzad Najafipour

**Affiliations:** 1 Nutrition Research Center, Tabriz University of Medical Sciences, Tabriz, Iran; 2 Endocrine Research Center, Tabriz University of Medical Sciences, Tabriz, Iran

**Keywords:** Hypothyroidism, Insulin resistance, Levothyroxine, Insulin, HOMA-IR

## Abstract

**Objectives:**

This study investigated the effects of levothyroxine replacement therapy on insulin resistance, lipid profile, and thyroid function in patients with untreated primary hypothyroidism. 105 patients with hypothyroidism with indication for levothyroxine replacement were enrolled in the present study. Insulin, fasting blood glucose and lipid profile were assessed at the beginning of diagnosis and three months after levothyroxine replacement. Insulin resistance was calculated by hemostasis model assessment of insulin resistance (HOMA-IR) and quantitative insulin sensitivity check index (QUICKI).

**Results:**

Our data revealed a significant reduction in body mass index (27.18 ± 4.27 versus 26.81 ± 4.18 kg/m2, p = 0.028), cholesterol (199.79 ± 37.61 versus 178.10 ± 32.25 mg/dl, p < 0.001), triglyceride (160.41 ± 71.86 versus 146 ± 61.11 mg/dl, p = 0.012), low density lipoprotein-cholesterol (123.54 ± 30.7 versus 107.08 ± 26.98 mg/dl, p < 0.001), fasting insulin (8.91 ± 3.92 versus 8.05 ± 2.65 mIU/l, p < 0.001), and thyroid stimulating hormone (47.47 ± 3.4 versus 2.22 ± 1.84 µIU/ml, p < 0.001) levels before and after drug intervention. However, no statistical differences were observed in HOMA-IR, QUICKI, and high density lipoprotein-cholesterol. In conclusion, in patients with untreated primary hypothyroidism, levothyroxine replacement therapy based on HOMA-IR and QUICKI did not improve insulin resistance; however, lipid profile was significantly improved following levothyroxine administration.

**Trial registration:**

This study was registered in the Iranian Registry of Clinical Trials (IRCT) with ID number: IRCT20130610013612N10 on the date 2019-09-02.

**Supplementary Information:**

The online version contains supplementary material available at 10.1186/s13104-023-06516-7.

## Introduction

Insulin resistance (IR) and the metabolic syndrome are complex metabolic traits defined as decreased response to insulin in tissues. Insulin is an endocrine peptide hormone which controls whole-body glucose homeostasis. It has been reported that both genetic and environmental factors are known to influence IR [1].

Thyroid hormones influence glucose homeostasis and the development of IR. They also upregulate genes such as glucose transporter type-4 (GLUT-4) and phosphoglycerate kinase that encode enzymes stimulating glycogenolysis and gluconeogenesis, thereby increasing the hepatic glucose production [2]. Recently, evidence has been shown that the sympathetic nerves can also stimulate hepatic glucose production, in addition to the direct effect of thyroid hormones [3]. Many studies have also reported the association between IR and hypothyroidism [2, 4, 5]. Hypothyroidism and hyperthyroidism constitute an IR state. However, the mechanism of each is different. So that, in hyperthyroidism, IR is predominant at the hepatic level and, in hypothyroidism, IR is at the level of peripheral tissues [6].

IR is the basis of metabolic syndrome pathophysiology, which is itself a risk factor for cardiovascular diseases (CVDs) [7]. It has been reported that subclinical hypothyroid with the thyroid stimulating hormone (TSH) levels ≥ 10 µIU/ml is associated with an increased risk of CVD and mortality [8].

The association between subclinical hypothyroidism and IR has also been shown in some previous studies [1, 5, 9]. However, other studies have not reported such relationship [10]. Mazaheri et al. [11] also compared IR in patients with Hashimoto’s hypothyroidism and hypothyroidism after thyroidectomy; however, no significant difference was observed between two groups.

Hormone replacement therapy in early hypothyroidism has also reduced IR in several studies [12, 13], although, other studies reported no significant effects in this regard [14, 15]. Thyroid hormones also play a significant role in 3-Hydroxy-3-methylglutaryl–coenzyme A (HMG-CoA) reductase induction as the main enzyme in cholesterol biosynthesis. Moreover, triiodothyronine (T3) hormone by inducing the low density lipoprotein-cholesterol (LDL-C) receptor gene plays a crucial role in LDL-C clearance from blood circulation [16, 17]. It has previously reported that total cholesterol and LDL-C levels are increased in patients with hypothyroidism [18]. Hence, hypothyroidism is a common cause of secondary dyslipidemia [19, 20]. Additionally, the activity of the lipoprotein lipase enzyme is reduced in hypothyroidism; therefore, hypothyroidism may be associated with higher triglyceride levels [21–23]. On the other hand, the association between hypothyroidism and ischemic heart disease has also been reported by several studies [24, 25]. Since, IR can be considered as the underlying cause in different diseases and there are controversial results regarding the association between thyroid hormone levels and IR, the present clinical trial was designed to estimate the effects of levothyroxine replacement therapy on IR, lipid profile, and thyroid function in patients with untreated primary hypothyroidism.

## Main text

### Materials and methods

#### Study setting

The present interventional clinical trial (IRCT20130610013612N10) was performed on patients diagnosed with hypothyroidism with levothyroxine treatment indication referred to the endocrinology clinic of Imam Reza Hospital and Sheikh Al-Reis Clinic at Tabriz University of Medical Sciences. This trial was approved by the Ethics Committee of Tabriz University of Medical Sciences, Tabriz, Iran (Ethics code: TBZMED. REC.1398.457). All patients signed an informed consent form at baseline. All methods were carried out in accordance with Consort guidelines and regulations.

Patients with primary hypothyroidism or previously untreated or uncontrolled hypothyroidism aged between 18 and 65 years were included in the present study. Subjects with metabolic syndrome could also participate in this study. Diabetes mellitus (fasting blood glucose (FBG) ≥ 126 mg/dL) or familial history of diabetes, heart failure, liver and kidney diseases and polycystic ovary syndrome, pregnancy, lactation and consumption of levothyroxine, statins and contraceptive pills during the last three months before the study were also considered as exclusion criteria.

Endocrinologist decided the doses of levothyroxine substitution and the target TSH was 0.5–4.5 miu/l. In participants, all parameters were assessed at the beginning of diagnosis and three months after levothyroxine treatment (1.7 mg/kg of body weight daily). This study adheres to CONSORT guidelines.

#### Sample size

A total of 105 patients were calculated using the mean (± standard deviation [SD]) of TSH obtained from the study of Nada AM [11], based on a confidence interval (CI) of 95% and power of 90% in 2-sided tests. We used the following statistical formula for calculation of sample size.

$$n = \frac{{{{({z_{1 - \alpha }} + {z_{1 - \beta }})}^2}{\sigma ^2}}}{{({\mu _0} - {\mu _1})^2}}$$

#### Blood sampling, biochemical measurements, and anthropometric measurements

After 12–14 h overnight fasting, the participants’ blood samples were collected and centrifuged at 3,000 rpm for 5 min to extract the serum. Serum FBG, total cholesterol (TC), high-density lipoprotein cholesterol (HDL-C), and triglyceride were measured using colorimetric-enzymatic methods using commercial kits (ParsAzmoon Co., Tehran, Iran). Low-density lipoprotein cholesterol (LDL-C) was calculated by the Friedewald equation. The enzyme-linked immunosorbent assay (ELISA) method was used to measure serum insulin using commercial kits (Monobind, Lake Forest, CA, USA). IR was also calculated by hemostasis model assessment of IR (HOMA-IR) and the quantitative insulin sensitivity check index (QUICKI). The serum TSH and free T4 (FT4) concentrations were measured by electrochemiluminescence assay.

After an overnight fasting, height and weight were measured to the nearest 0.1 cm and 0.1 kg, respectively, with light clothes and no shoes using a calibrated scale and stadiometer (Seca, Germany). Body mass index (BMI) was estimated as weight (Kg) divided by height squared (m^2^).

#### Statistical analysis

SPSS software (statistical package for social analysis, ver. 20, SPSS Inc., Chicago, IL, USA) was used for data analysis. Mean values were compared using paired T-test between groups. P < 0.05 was also considered as statistically significant.

## Results

Table 1 shows the characteristics of the study participants. In this interventional clinical trial study, 105 subjects (males = 34.28% and females = 65.72%) were enrolled. Metabolic syndrome was diagnosed in 61.11% of males and 63.76% of females (p = 0.791).

**Table 1:** Baseline characteristics of the study participants

Variable	Males (n=36)	Females (n=36)	p
Age (years)	41.65 ± 9.31	43.72 ± 10.33	0.320*
Weight (kg)	84.33 ± 15.27	79.56 ± 11.23	0.078*
BMI (Kg/m2)	25.32 ± 7.56	26.11 ± 6.98	0.601*
Metabolic syndrome (n%)	22 (61.11)	44 (63.76)	0.791**
Obesity	17 (47.22)	29 (42.02)	0.610 **
High waist circumference	23 (63.88)	45 (65.21)	0.894**
Hypertension	21 (58.33)	31 (44.92)	0.193**
High triglyceride	19 (52.77)	29 (42.02)	0.295**
Low HDL-C	17 (47.22)	33 (47.82)	0.950 **

Data are presented as mean ± standard deviation or number (%). HDL-C, high-density lipoprotein cholesterol

Obesity: Body mass index (BMI) ≥ 30 kg/m2

High waist circumference: >102 cm in men and > 88 cm in women

High triglycerides: ≥ 150 mg/dL

Low HDL-C: <40 mg/dL in men and < 50 mg/dL in women

Hypertension: systolic blood pressure ≥ 130 mm Hg or diastolic blood pressure ≥ 85 mm Hg

* Independent samples t-test

** Chi-square test

Table 2 shows BMI, biochemical, IR indices and thyroid function tests (TFT) before and after levothyroxine administration. Significant differences were observed in BMI (27.18 ± 4.27 versus 26.81 ± 4.18 kg/m2, p = 0.028), cholesterol (199.79 ± 37.61 versus 178.10 ± 32.25 mg/dl, p < 0.001), triglyceride (160.41 ± 71.86 versus 146 ± 61.11 mg/dl, p = 0.012), LDL-C (123.54 ± 30.7 versus 107.08 ± 26.98 mg/dl, p < 0.001), fasting insulin (8.91 ± 3.92 versus 8.05 ± 2.65 mIU/l, p < 0.001), TSH (47.47 ± 3.4 versus 2.22 ± 1.84 µIU/ml, p < 0.001) and FT4 (0.8 ± 0.19 versus 1.20 ± 0.44 ng/dl, p < 0.001) before and after drug intervention. No significant differences were observed regarding the HDL-C, FBG, HOMA- IR and QUICKI.

**Table 2:** Biochemical parameters, insulin resistance and thyroid function tests before and after levothyroxine treatment

Parameters		Before	After	p*
**Anthropometric**	**Weight (Kg)**	73.45± 9.33	68.25±7.77	**0.001**
	**BMI (kg/m** ^**2**^ **)**	27.13±4.27	26.81±4.18	**0.028**
**Biochemical**	**Cholesterol (mg/dl)**	199.79±37.61	178.10±32.25	**<0.001**
	**Triglyceride (mg/dl)**	160.41±71.86	146±61.11	**0.012**
	**HDL-C (mg/dl)**	44.31±10.56	44.31±10.59	0.954
	**LDL-C (mg/dl)**	123.54±30.7	107.08±26.98	**<0.001**
**IR**	**FBG (mg/dl)**	91.95±13.17	92.69±13.38	0.134
	**Fasting insulin (mIU/l)**	8.91±3.92	8.05±2.65	**<0.001**
	**HOMA-IR**	2.07±1.56	2.09±1.46	0.451
	**QUICKI**	0.356±0.36	0.355±0.035	0.934
**TFT**	**TSH (µIU/ml)**	47.45±3.4	2.22±1.84	**<0.001**
	**FT4 (ng/dl)**	0.8±0.19	1.20±0.44	**<0.001**

Data are presented as mean ± standard division. P < 0.05 was considered as statistically significant. BMI, body mass index; HDL, high-density lipoprotein cholesterol; LDL-C, low-density lipoprotein cholesterol; FBG, fasting blood glucose; HOMA-IR, homeostatic model assessment for insulin resistance; QUICKI, quantitative insulin sensitivity check index; IR, insulin resistance; TFT, thyroid function test

*Paired samples t-test

P < 0.05 is statistically significant

## Discussion

In the literature, there are two main non-invasive (HOMA-IR and QUICKI) [26] and invasive (hyperinsulinemic-euglycemic clamp technique) methods for IR assay [27]. In the present study, HOMA-IR and QUICKI were used to evaluate IR. Our data revealed an increased basal HOMA-IR level which was in line with the study of Estaghamati et al. [28] with the estimated 1.78 HOMA-IR level.

After receiving levothyroxine and euthyroidism induction, the rate of IR in our patients did not significantly change; although, a significant decreased BMI level was reached among patients. Similarly, study of Brenta et al. [10] showed that treatment with levothyroxine did not reduce the IR. Teixeira et al. [18, 29] also showed no improvement in IR and BMI levels after levothyroxine treatment. However, Stanicka et al. [8] and Handisurya et al. [9] reported a significant decrease in IR after levothyroxine treatment. Similar results were also reported by Kowalska et al. [15], Deyneli et al. [17] and Rochon et al. [20] regarding the improved IR after levothyroxine administration. In another study conducted by Brenta et al. [16], decreased IR was also observed after this drug administration which all mentioned studies used hyperinsulinemic-euglycemic clamp technique to evaluate IR. Generally, in patients with hypothyroidism before and after levothyroxine replacement therapy, no significant changes in IR have been reported using HOMA-IR as IR indicator which are in line with our finding [10, 11, 18]. However, in studies using hyperinsulinemic-euglycemic clamp technique to evaluate IR, levothyroxine replacement has led to significant reduction in IR [8, 9, 15–17, 20]. Hypothyroidism causes peripheral IR, and hyperinsulinemic-euglycemic clamp technique is the more exact method for its evaluation. However, the number of studies using this technique is limited because of its difficulties and side effects.

In study of Stanicka et al. [8], despite non-significant change in basal insulin levels and C-peptide, by glucose disposal rate determination, a decreased IR has been reported following levothyroxine replacement, and due to the increase in other examined hormones, they concluded that hypothyroidism may increase IR with the other mechanisms such as increased counter regulatory hormones levels. In other words, IR is usually observed as impaired glucose tolerance, and increased blood glucose and insulin levels after meals or after glucose tolerance testing are likely to have more accurate results than that of blood glucose and fasting insulin levels which are used in HOMA-IR and QUICKI tests.

Different studies have shown that levothyroxine replacement significantly improves lipid metabolism disorders, and a treatment period of 4 to 6 weeks is needed to correct dyslipidemia in hypothyroidism. The rate of dyslipidemia improvement is related to FT4 levels. Recent studies in hypothyroidism patients have also shown decreased serum total cholesterol and LDL-C levels after levothyroxine treatment.

In our study, thyroid hormones replacement improved dyslipidemia in patients. Levothyroxine replacement therapy in hypothyroid patients has previously been shown to improve cholesterol and LDL-C levels and increase HDL-C levels. In our study, levothyroxine treatment also caused a significant decrease in cholesterol, triglyceride and LDL-C levels. In the study of Brenta et al. [10] levothyroxine treatment did not correct the basal lipid derangement. In contrast, in a study conducted by Aml Mohamed Nada [11], a significant decrease in cholesterol level was observed after levothyroxine treatment; however, triglyceride level did not alter significantly. Deyneli et al. [17] also reported decreased cholesterol and LDL-C levels after levothyroxine administration.

### Limitations

The limitations of our study were a small number of hypothyroid patients and the loss of nutritional and lifestyle data. Given that our study included a small number of subjects, we were unable to completely show the effects of replacement therapy. Moreover, our study duration was short; longer periods might reveal different results. Future studies with larger sample sizes and longer duration are recommended.

## Conclusion

In the present study, levothyroxine replacement therapy in hypothyroid patients led to significant improvements in lipid profile and BMI. However, levothyroxine replacement therapy based on HOMA-IR and QUICKI methods did not improve IR. Since, mentioned methods may not have the enough sensitivity for evaluation of T4 therapy in mild IR cases, other tests with higher sensitivity are proposed.

### Electronic supplementary material

Below is the link to the electronic supplementary material.

Supplementary Material 1

## Data Availability

The datasets used and/or analyzed during the current study are available from the corresponding author on reasonable request.
